# The Prevalence of Malaria among Pregnant Women in Ethiopia: A Systematic Review and Meta-Analysis

**DOI:** 10.1155/2019/8396091

**Published:** 2019-05-02

**Authors:** Yalewayker Tegegne, Daniel Asmelash, Sintayehu Ambachew, Setegn Eshetie, Ayenew Addisu, Ayalew Jejaw Zeleke

**Affiliations:** ^1^Department of Medical Parasitology, School of Biomedical and Laboratory Sciences, College of Medicine and Health Sciences, University of Gondar, Gondar, Ethiopia; ^2^Department of Clinical Chemistry, School of Biomedical and Laboratory Sciences, College of Medicine and Health Sciences, University of Gondar, Gondar, Ethiopia; ^3^Department of Medical Microbiology, School of Biomedical and Laboratory Sciences, College of Medicine and Health Sciences, University of Gondar, Gondar, Ethiopia

## Abstract

**Background:**

Malaria during pregnancy remains a major public health concern in tropical and subtropical countries. Moreover, malaria is increasingly associated with unwanted pregnancy outcomes such as an increased risk of abortion, stillbirth, premature delivery, and low-birthweight infants. Since pregnant women are most vulnerable to malaria, implementation of the appropriate prevention and control measures among this group is very important. Therefore, the current review was designed to assess the prevalence of both symptomatic and asymptomatic malaria among pregnant women in Ethiopia.

**Method:**

In this systematic review and meta-analysis we have followed Preferred Reporting Items for Systematic Reviews and Meta-Analyses (PRISMA) guideline. The databases used were PubMed, Google Scholar, HINARI, and Science Direct literature. Search terms used were “prevalence”, “malaria”, “pregnant women”, and “Ethiopia”. Joanna Briggs Institute Meta-Analysis of Statistics Assessment and Review Instrument (JBI-MAStARI) was used for critical appraisal of studies. The meta-analysis was conducted using STATA 14 software. The pooled meta-logistic regression was computed to present the pooled prevalence with a 95% confidence interval (CI).

**Result:**

Among a total of 10207 studies, seven studies were included in this analysis. The estimated pooled prevalence of malaria among pregnant women in Ethiopia was 12.72% (95% CI: 7.45, 17.98). In subgroup analysis, the prevalence of malaria showed a significant variation between asymptomatic and symptomatic cases, which was 7.83% (95% CI: 2.23, 13.43) and 17.97% (95% CI: 7.31, 28.92), respectively.

**Conclusion:**

The current systematic review and meta-analysis showed that the pooled prevalence of malaria among pregnant women was found to be relatively higher compared with the general population. Therefore, the existing prevention and control measures should be strengthen.

## 1. Background

In malaria endemic areas, pregnant women are the highest risk group for malaria infection and to develop a severe form of the disease that results in mortality. Thus, increasing the use of antimalaria interventions that target pregnant women which can address the social, cultural, and economic factors that heighten susceptibility has the potential to control the disease in most of the susceptible and underserved groups [1].

Infection of malaria during pregnancy is common, which can result in fetus low birthweight, stillbirth, and decrement in intrauterine fetal growth. Besides, malaria infection has the greatest impact on the survival of mothers. The factor behind the high burden of malaria during pregnancy could be the increased body surface and specific odor secretions during pregnancy which may expose them to increased mosquito bites [2, 3].

Malaria is one of the killer diseases worldwide. According to the World Health Organization (WHO) report in 2016, around 216 million new cases of malaria occurred globally. Besides, most of the malaria cases were in the African region (90%) followed by the Southeast Asia region (7%) and Eastern Mediterranean region (2%). Similarly, there was an estimated 445000 malaria deaths worldwide. Most of these deaths occurred in the Africa region (91%) followed by the Southeast Asia region (6%) and Eastern Mediterranean region (2%) [4]

According to the Ethiopian Federal Minster of Health (FMH) report, approximately 68% of the Ethiopian people live in malaria risk areas, around 75% of the landmass of Ethiopia is malaria endemic, and it is one of the most malaria prone countries in Africa. On average, 60%-70% of malaria cases have been due to* P. falciparum*, with the remainder caused by* P. vivax*.* Anopheles arabiensis* is the main malaria vector;* An. pharoensis*,* An. funestus*, and* An. nili* play a role as secondary vectors [5].

Malaria infection during pregnancy is a major public health concern in tropical and subtropical countries with significant risk for the pregnant woman and her fetus. According to the estimated yearly report, the number of pregnant women who were at risk of malaria was about 25 million. It has been reported that in sub-Saharan Africa malaria can cause as many as 10,000 cases of malaria-related deaths in pregnancy per year, usually due to severe maternal anemia [6]. Besides, each year, malaria in pregnancy is responsible for 20% of stillbirths, and 11% of all newborn deaths in sub-Saharan Africa [7].

The rate of malaria infection is higher in pregnant women because of their decreased immunity. Mainly pregnant women living in areas of low or unstable malaria transmission have little or no immunity to malaria and are at higher risk of developing the severe disease as a result of malaria infection than nonpregnant adults living in the same area. Pregnant women with malaria have an increased risk of abortion, stillbirth, premature delivery, and low-birthweight infants [8, 9]. Moreover, in unstable malaria transmission areas, pregnant mothers death may be due to complications of severe malaria (hypoglycaemia, cerebral malaria, and pulmonary edema) or indirectly from malaria-related severe anemia [9].

Despite this fact, studies conducted to assess the prevalence rate of malaria among pregnant women in Ethiopia have a great disparity and inconsistent findings. Moreover, there is no previous systematic review and meta-analysis that estimated the prevalence rate of malaria among pregnant women in Ethiopia. Therefore, the current systematic review and meta-analysis was designed to assess the prevalence of malaria among pregnant women in Ethiopia. This helps in the implementation of the most substantial malaria prevention and control measures.

Currently, the most substantive malaria prevention and control measures include insecticide-treated mosquito nets (ITNs), indoor residual spraying (IRS), intermittent preventive treatment for pregnant women (IPTp), seasonal malaria chemoprevention (SMC), and diagnosis by malaria microscopy or rapid diagnostic test (RDT), together with effective treatment for confirmed malaria cases with artemisinin-based combination therapies (ACTs) [10]. Thus, this review is aimed at determining the pooled prevalence of malaria among pregnant women in Ethiopia.

## 2. Methods

### 2.1. Search Strategy

This systematic review and meta-analysis was reported according to the Preferred Reporting Items for Systematic Reviews and Meta-Analyses (PRISMA) statement guideline. Pertinent published articles were searched in the following electronic bibliographic databases: PubMed, Google Scholar, HINARI, and Science Direct literature, to identify studies conducted on the prevalence of malaria among pregnant women and published up to August 2018. The search terms were used in agreement with the Medical Subject Heading (MeSH) using the arrangement of key words which were used to select relevant studies. The search terms were used separately and in combination using Boolean operators like “OR” or “AND”. An example of search strategy used to retrieve relevant articles was as follows: ((((((((prevalence) OR prevalence [MeSH Terms]) AND malaria) OR malaria [MeSH Terms]) AND pregnant women) OR pregnant women [MeSH Terms]) AND Ethiopia) OR Ethiopia [MeSH Terms]). Duplicate data were excluded. The software EndNote version X7 (Thomson Reuters, New York, NY) was used to manage references and remove duplicated references.

### 2.2. Eligibility Criteria

#### 2.2.1. Inclusion Criteria

Studies which have been published in different peer-reviewed journals on the prevalence of malaria among pregnant women were included. All studies were original research published in English and contained the minimum information concerning sample size and status of malaria infection, which helps to analyze a pooled estimate of the prevalence of malaria among pregnant women in Ethiopia. Besides, studies in which malaria has been diagnosed using microscopy, rapid diagnostic test, and polymerase chain reaction were included. Moreover, studies which have been carried out in Ethiopia among pregnant women attending ANC (Anti Natal Clinic) and community-based studies among pregnant women were also included. Studies which were done on symptomatic or asymptomatic pregnant women were included in this systematic review.

#### 2.2.2. Exclusion Criteria

Studies done among women in the delivery unit, unknown methods of malaria diagnosis, and questionnaire-based studies which were intended to assess the burden of malaria among pregnant women were excluded.

### 2.3. Search Methods and Quality Assessment

Six authors (YT, SA, SE, DA, AA, and AJZ) independently conducted a search in PubMed, Google Scholar, HINARI, and Science Direct literature, using the key words, for including researches which were published up to August 2018. Then searched articles were screened by the title and abstract to consider the articles in the full-text review. Following exclusion of duplicates, abstracts and titles of 125 papers were screened for eligibility criteria, and seven were chosen for full-text evaluation. Differences in the selection of articles being included in the review were resolved by the third reviewer decision, though there was a very low degree of discrepancy between the authors in the choice of articles for the review. The quality of articles was assessed using Joana Brigg's Institute (JBI) critical appraisal checklist for simple prevalence [11].

### 2.4. Data Extraction

An established data extraction tool, Microsoft Excel, was used for the data extraction. This extraction tool included evidence regarding the name of the author/s, publication year, study period, study design, sample size, the prevalence of malaria, the prevalence of* Plasmodium falciparum*, the prevalence of* Plasmodium vivax*, the prevalence of mixed infection, and the type of malaria laboratory diagnostic method used. Preferred Reporting Items for Systematic Reviews and Meta-Analyses (PRISMA) guideline was strictly followed when conducting this review.

### 2.5. Data Analysis

Eligible primary studies data were extracted, entered into Microsoft Excel, and then exported to STATA version 14. Moreover, in this study, forest plots were used to estimate pool effect size and effect of each study with their confidence interval (CI) to provide a visual image of the data. The degree of heterogeneity between the included studies was evaluated by the index of heterogeneity (I^2^ statistics)[12]. I^2^ values of 25%, 50%, and 75% are assumed to represent low, medium, and high heterogeneity, respectively. The analysis between the subgroups was carried out regarding the type of cases. Small study effect and publication bias were evaluated by funnel plot test. Besides, study bias was evaluated using Egger's and Begg's test.

## 3. Result

### 3.1. Characteristics of Included Studies

**Figure 1:**
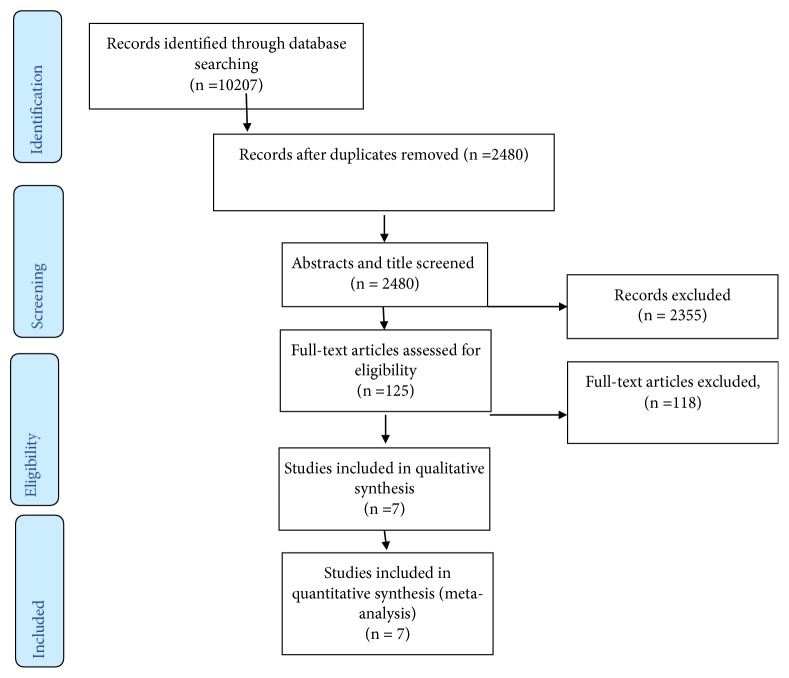
Flow chart to describe the selection of studies for the systematic review and meta-analysis of the prevalence of malaria among pregnant women.

This systematic review includes published papers on the prevalence of malaria among pregnant women. After searching for available publications, initially we found a total of 10207 published articles. From this, 7727 duplicate records were removed, 2355 records were excluded after screening by title and abstracts, and 125 were found to be eligible for full-text assessment. Among the total full-text screened articles, only seven full-text articles were screened for eligibility. Finally, all the 7 studies were included in the final quantitative meta-analysis (Figure 1).

All of the studies which were included in this systematic review and meta-analysis had cross-sectional study design and were conducted among pregnant women. The minimum sample size was 87 participants in a study conducted in Gondar [13], while the highest sample size was 760, in Amhara, Oromia, and Gambella regions [14]. The overall number of study participants who were included in this meta-analysis was 2842 pregnant women. The majority of the studies were conducted among pregnant women with symptomatic malaria, 4 (57%), and the rest of the studies were conducted among pregnant women with asymptomatic malaria, 3 (43%). In this systematic review and meta-analysis, most of the regions were included. When we look at the study sites, we find that one of the included studies was conducted in Adama, a city in the Oromia region of Ethiopia [15], two in Amhara region [13, 16], two in Southern Nation Nationality People Republic of Ethiopia [17, 18], and nationwide one in the three regions of Oromia, Amhara, and Benishangul-Gumuz [14] (Table 1).

### 3.2. Prevalence of Malaria among Pregnant Women

**Figure 2:**
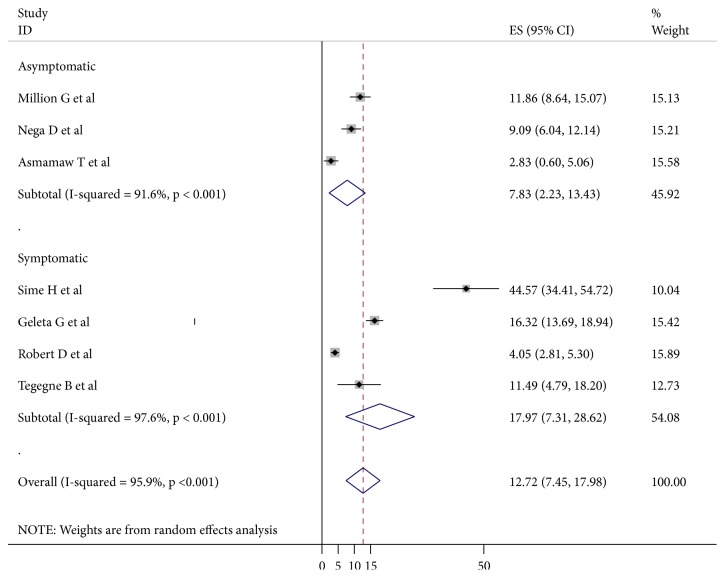
The pooled estimates of the prevalence of malaria among pregnant women from random effect model by type of cases. The midpoint and the length of each segment indicated prevalence and a 95% CI, whereas the diamond shape showed the combined prevalence of all studies.

Seven published studies were included in this systematic review and meta-analysis and all of these studies were used to estimate the pooled prevalence of malaria among pregnant women. The minimum prevalence of malaria was 2.8% and it was found in Amhara region of Felege Hiwot Referral Hospital, Bahir Dar, and Addis Zemen Health Center [16]. On the other hand, the maximum malaria prevalence was found to be 44.6%, in a study conducted in Central Ethiopia, Adama [19]. The* I*^*2*^ test result showed high heterogeneity (I^2^ 95.9%,* P* = <0.000). Using the random effect analysis, the pooled prevalence of malaria among pregnant women in Ethiopia was 12.72% (95% CI (7.45, 17.98)). Subgroup analysis based on types of malaria cases showed that the prevalence of malaria among asymptomatic and symptomatic pregnant women was found to be 7.83% (95% CI: 2.23, 13.43) and 17.97% (95% CI: 7.31, 28.92), respectively (Figure 2).

### 3.3. Prevalence of Malaria among Pregnant Women by Species of Plasmodium Parasites

**Figure 3:**
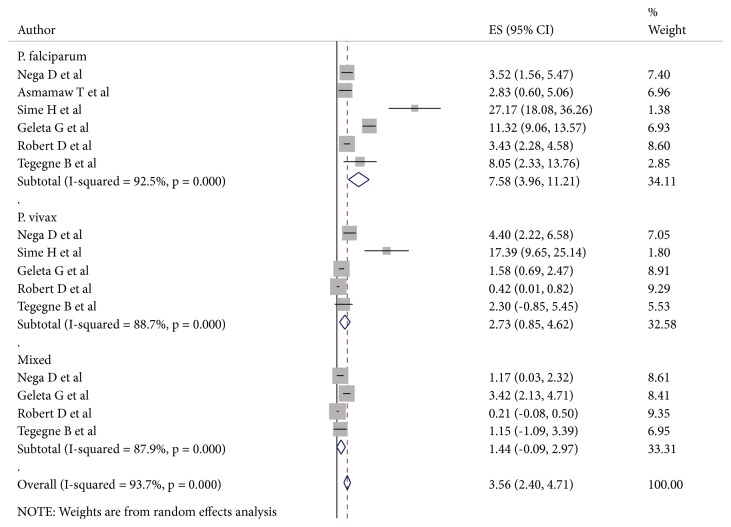
The pooled estimates of the prevalence of malaria among pregnant women from random effect model by the type of* Plasmodium* species. The midpoint and the length of each segment indicated prevalence and a 95% CI, whereas the diamond shape showed the combined prevalence of all studies.

The prevalence of* Plasmodium* parasite species among Ethiopian pregnant women was compared between studies. From all of the studies, only five have reported the prevalence of* P. vivax* among pregnant women. In addition, six of the seven studies have reported the prevalence of* P. falciparum* and only four of the total studies have reported the prevalence of mixed infection. The overall weighted pooled prevalence of* Plasmodium* species was as follows:* P. vivax* 2.74 (95% CI: 0.85,4.62),* P. falciparum* 7.58 (95% CI: 3.96,11.20), and mixed infection 1.44 (95% CI: -0.08,2.96) (Figure 3).

### 3.4. Sensitivity Analysis

We did the sensitivity analysis of the prevalence of malaria among pregnant women by applying a random effects model (Table 2). The analysis was done to evaluate the effect of each study on the pooled estimated prevalence of malaria by excluding each study step-by-step. The result showed that excluded studies did not show significant difference in the prevalence of malaria among pregnant women.

### 3.5. Publication Bias

**Figure 4:**
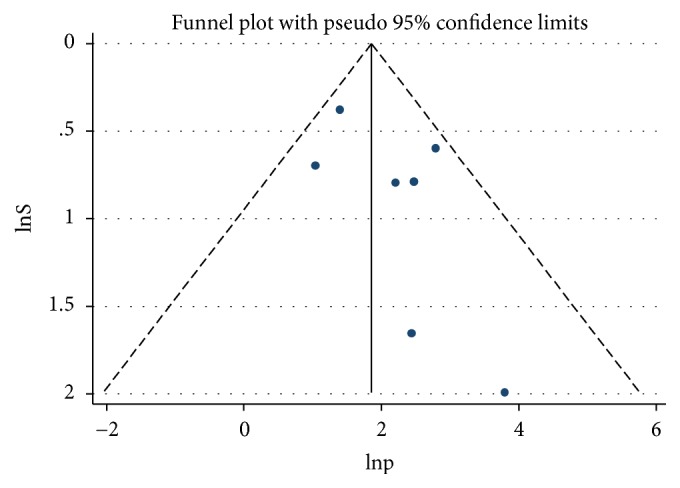
Funnel plot of the prevalence of malaria among pregnant women.

The included studies were assessed for potential publication bias visually by funnel plot. The funnel plot symmetrically indicated the absence of publication bias since greater than 100% of the studies fell within the triangular region (Figure 4). Besides, the result of Egger's test indicated no publication bias,* P*-values >0.05 (Table 3).

## 4. Discussion

The current systematic review and meta-analysis was carried out to determine the pooled prevalence of malaria among pregnant women in Ethiopia using seven studies, which were published in scientific and reputable journals. Malaria during pregnancy is associated with the reduced birthweight of infants, maternal anemia, maternal death, and infant mortality [20, 21]. Moreover a number of recently published studies have shown that malaria in pregnancy is associated with increases in fetal growth restriction, as a major determinant of infant mortality and increased risk of maternal morbidity and mortality [22, 23].

In this systematic review and meta-analysis the pooled prevalence of malaria among pregnant women in Ethiopia was 12.72 (95% CI: 7.45, 17.98). This is higher than the 2011 malaria indicator survey result which showed 1.3% prevalence of malaria among the general population of the country [24]. The reason behind the high prevalence of malaria among pregnant women could be their decreased immunity and physiological changes.

This systematic review and meta-analysis result was much lower than a previous systematic review carried out in West and Central Africa, which reported the prevalence rate of 38.2% [25]. The decrease in the prevalence of malaria in the current study might be related to life style and geographical area and might have been related to the malaria elimination and control program which has been launched in Ethiopia.

The prevalence rate of malaria among pregnant women has shown difference in various part of the world like India (27%), Rwanda 13.6%[26], Uganda 87.9%[27], and Sudan 56.5% [28]. The prevalence of malaria in this study was lower than the above-mentioned studies; the difference might be related to economic status, increased awareness of pregnant women regarding prevention and control measures of malaria, time of study conducting, and the type of malaria laboratory diagnosis method being used.

Moreover, as per the WHO recommendation in Ethiopia, malaria prevention and control measures, like proper use of long lasting insecticide-treated nets (LLIN), insecticide residual spraying (IRS), introduction of rapid diagnostic tests at community level, and adaptation of artemisinin-based combination therapies (ACTs), have been utilized, which might have led to reduction in the burden of malaria in Ethiopia [29].

Subgroup analysis based on the type of cases showed a lower and higher prevalence of malaria among asymptomatic pregnant (7.8%) women and malaria symptomatic (17.97%) pregnant women, respectively. The difference in the prevalence of malaria between types of cases could be due to the fact that, in symptomatic malaria cases, there might be higher parasite load that can be detected in the laboratory.

The highest and lowest prevalence of malaria was observed in study done in Adama, Central Ethiopia (44.6%), and in Felege Hiwot Referral Hospital, Bahir Dar, and Addis Zemen Health Center (2.8%). This could be explained by the existing differences in the environmental condition, rain fall, climate condition, residents' lifestyles, and applied prevention and control measures.

More than one reviewer was involved in this systematic review and meta-analysis, and we used a comprehensive search strategy. Moreover, during this review we have also strictly followed the PRISMA guideline. In this systematic review and meta-analysis, we encountered some limitations. For example, the number of studies which are included is limited, which might affect the pooled estimate of malaria prevalence among pregnant women in Ethiopia. All studies incorporated in this systematic review and meta-analysis were cross-sectional studies and the outcome variability may be affected by other confounding variables. These limitations might affect the results reported in this review regarding the overall prevalence of malaria in Ethiopia.

## 5. Conclusion

The current systematic review and meta-analysis showed that the pooled prevalence of malaria among pregnant women was found to be relatively higher compared with the general population. Therefore, the existing prevention and control measures should be strengthened, for example, health education regarding proper usage of LLIN; application of IRS for controling the vector and early diagnosis and treatment of pregnant women should be adopted. Moreover, for further accurate estimation of the burden of malaria among this vulnerable group, a national study that covers the whole region should be conducted in Ethiopia.

## Figures and Tables

**Table 1:** General characteristics and outcomes of the included studies (n=7).

Author	Study design	Region	Year of study	Type of cases	Sample size	Prevalence of malaria (%)	Study quality

Million G et al.	Cross sectional	Southwest Ethiopia	2009	Asymptomatic	388	11.86	Good
Nega D et al., 2015	cross sectional	South Ethiopia	2015	Asymptomatic	341	9.1	Good
Asmamaw T et al., 2013	cross sectional	Northwest Ethiopia	2013	Asymptomatic	212	2.8	Good
Sime H et al., 2009	cross sectional	Central Ethiopia	2009	Symptomatic	92	44.6	Good
Geleta G et al., 2017	cross sectional	Northwest Ethiopia	2017	Symptomatic	760	16.3	Good
Robert D et al.	cross sectional	Central Ethiopia	2003	Symptomatic	962	4	Good
Tegegne B et al., 2017	cross sectional	Northwest Ethiopia	2017	Symptomatic	87	11.4	Good

**Table 2:** Sensitivity analysis of the included studies to estimate the pooled prevalence of malaria among pregnant women.

Study omitted	Prevalence of malaria among pregnant women (95% CI)

Million G et al., 2009	12.98(9.46-19.74)
Nega D et al., 2015	12.57(9.37-39.12)
Asmamaw T et al., 2013	13.57(9.37-39)
Sime H et al., 2009	9.1(4.5-13.6)
Geleta G et al., 2017	11.7(6.5-16.8)
Robert D et al., 2003	14.67(8.08-21.26)
Tegegne B et al., 2017	12.93(7.2-18.6)

**Table 3:** Egger's test.

Std_Eff	Coef.	Std. Err.	T	P>t	[95% Conf.	Interval]

slope	-1.524136	.8535549	-1.79	0.134	-3.718269	.669997
bias	-2.647537	5.073598	-0.52	0.624	-15.68964	10.39456

## Data Availability

The main part of the data generated or analyzed during this study is included in this published article. Other data will be available from the corresponding author upon request.

## Abbreviations

ACTs: Artemisinin-based combination therapies
ANC: Anti Natal Clinic
FMH: Federal Minster of Health
IRS: Indoor residual spraying
ITNs: Insecticide-treated mosquito nets
IPTp: Intermittent preventive treatment for pregnant women
JBI: Joanna Brigg's Institute
PRISMA: Preferred Reporting Items for Systematic Reviews and Meta-Analyses
RDT: Rapid diagnostic test
WHO: World Health Organization.
